# Differential and antagonistic effects of 9- *cis* -retinoic acid and vitamin D analogues on pancreatic cancer cells in vitro

**DOI:** 10.1054/bjoc.2000.1281

**Published:** 2000-06-15

**Authors:** F Pettersson, K W Colston, A G Dalgleish

**Affiliations:** Department of Oncology, Gastroenterology, Endocrinology and Metabolism, St George’s Hospital Medical School, London, SW17 0RE, UK

**Keywords:** 9- *cis* -retinoic acid, vitamin D analogues, pancreatic cancer, growth inhibition, apoptosis

## Abstract

Retinoids and vitamin D are known to exert important anti-tumour effects in a variety of cell types. In this study the effects of 9- *cis* -retinoic acid (9cRA) the vitamin D analogues EB1089 and CB1093 on three pancreatic adenocarcinoma cell lines were investigated. All compounds caused inhibition of in vitro growth but the vitamin D analogues were generally the more potent growth inhibitors. They were also more effective on their own than in combination with 9cRA. Growth arrest correlated with an increased proportion of cells in the G0/G1 phase. Apoptosis was induced in the three cell lines by 9cRA, whereas neither EB1089 nor CB1093 had this effect. Furthermore, addition of EB1089 or CB1093 together with 9cRA resulted in significantly reduced apoptosis. Our results show that retinoic acids as well as vitamin D analogues have inhibitory effects on pancreatic tumour cells but different and antagonistic mechanisms seem to be employed. © 2000 Cancer Research Campaign


*British Journal of Cancer* (2000) **83**(2), 239–245
© 2000 Cancer Research Campaign
doi: 10.1054/ bjoc.2000.1281, available online at http://www.idealibrary.com on IDEAL®

### Differential and antagonistic effects of 9-*cis*-retinoic acid and vitamin D analogues on pancreatic cancer cells in vitro

**F Pettersson, KW Colston and AG Dalgleish**

Department of Oncology, Gastroenterology, Endocrinology and Metabolism, St George's Hospital Medical School, London SW17 0RE, UK

**Summary** Retinoids and vitamin D are known to exert important anti-tumour effects in a variety of cell types. In this study the effects of 9-*cis*-retinoic acid (9cRA) the vitamin D analogues EB1089 and CB1093 on three pancreatic adenocarcinoma cell lines were investigated. All compounds caused inhibition of in vitro growth but the vitamin D analogues were generally the more potent growth inhibitors. They were also more effective on their own than in combination with 9cRA. Growth arrest correlated with an increased proportion of cells in the G0/G1 phase. Apoptosis was induced in the three cell lines by 9cRA, whereas neither EB1089 nor CB1093 had this effect. Furthermore, addition of EB1089 or CB1093 together with 9cRA resulted in significantly reduced apoptosis. Our results show that retinoic acids as well as vitamin D analogues have inhibitory effects on pancreatic tumour cells but different and antagonistic mechanisms seem to be employed. © 2000 Cancer Research Campaign

**Keywords**: 9-*cis*-retinoic acid; vitamin D analogues; pancreatic cancer; growth inhibition; apoptosis

Adenocarcinoma of the pancreas is one of the most common causes of cancer deaths in the Western world. At the time of diagnosis, the vast majority of patients have advanced disease and the tumour is no longer respectable. For many years, 5-fluorouracil has been the standard chemotherapeutic drug used in the treatment of pancreatic cancer, and more recently gemcitabine has shown promising activity (Rothenberg et al, 1996) and is gaining widespread use. However, response rates fail to exceed 20% even with combination therapy (Evans et al, 1996; Dippold et al, 1997) and neither radiation nor chemotherapy significantly improve long-term survival (Gansauge et al, 1996; Rosewicz and Wiedenmann, 1997). The dismal prognosis of this disease makes it an appropriate model for evaluation of new, alternative therapies such as treatment with differentiating agents like retinoids and vitamin D analogues.

Retinoids, which are derivatives of vitamin A, are important factors involved in the control of many biological functions, including cell growth and differentiation, development and carcinogenesis. The main function of the active metabolite of vitamin $D_3$, 1,25-dihydroxyvitamin $D_3$ (1,25$D_3$), is as a regulator of serum calcium, although it also has effects on normal and malignant tissue similar to those of the retinoids. Studies of the anti-tumour actions of retinoids and vitamin D derivatives show that growth inhibition, cellular differentiation and/or apoptosis can be induced in a variety of cell types (Bollag et al, 1994; Lotan, 1996; Nagy et al, 1998). A major limitation to the use of 1,25$D_3$ in a therapeutic setting is its potent induction of hypercalcaemia, and to circumvent this problem synthetic analogues with a more favourable profile of activity have been developed. Two of several that have been extensively characterized are EB1089 (Mathiasen et al, 1993) and CB1093 (Danielsson et al, 1997) (Figure 1). They both have more potent growth inhibitory effects than 1,25$D_3$ and EB1089 is also significantly less hypercalcaemic in vivo (Colston, 1997).

Most pancreatic cancer cells express the vitamin D receptor (VDR) (Kawa et al, 1996; Colston et al, 1997) as well as receptors for the retinoic acids (Rosewicz et al, 1995), suggesting that these cells may respond to treatment with these agents.

The retinoids and 1,25$D_3$ and its analogues exert most of their effects by binding to specific receptors belonging to the nuclear receptor superfamily. These receptors act as ligand-dependent transcription factors, switching a variety of genes on and off (Evans, 1988). There are two families of receptors binding the retinoic acids: the retinoic acid receptors (RARs) and the retinoid X receptors (RXRs). Each family has three subtypes, $\alpha$, $\beta$ and $\gamma$, and each of those a number of isoforms. This fact, together with differential expression of the receptor subtypes in various tissues and cell types, results in a board spectrum of biological activity being affected by the retinoids in vivo.

The natural ligand for RARs is all-*trans*-retinoic acid (ATRA), whereas the stereoisomer 9cRA binds to both RARs and RXRs with high affinity (Giguere et al, 1987; Petkovich et al, 1987; Heyman et al, 1992; Levin et al, 1992). RXRs also play a very important role in heterodimerization with other nuclear receptors such as VDR and RARs. All nuclear receptors bind as dimers to specific *cis*-acting hormone response elements (HREs) on the genes they control. VDR and RARs can bind to DNA as homodimers but for high affinity binding they require the presence of RXRs. This forms the molecular basis for interactions between retinoids binding to RXRs and vitamin D analogues (Kliewer et al, 1992; Haussler et al, 1995). In many cells the two groups of compounds affect growth and differentiation in similar ways. However, studies of the effects of 9cRA in combination with

*Received 9 November 1999*
*Revised 21 February 2000*
*Accepted 28 February 2000*

*Correspondence to:* F Pettersson

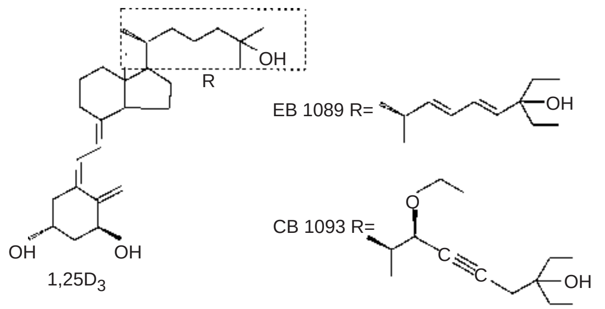

**Figure 1** Structures of 1,25-dihydroxyvitamin $D_3$ and the analogues used in the study

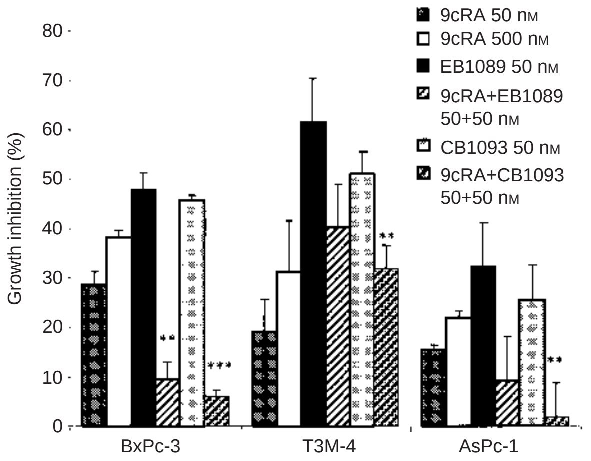

**Figure 2** All compounds tested induced inhibition of growth in AsPc-1, BxPc-3 and T3M-4 cells. Total cell numbers were assessed on day 6 using SRB staining and % inhibition was calculated compared to untreated control cultures. Results are expressed as means ± standard deviations of three separate experiments. Statistical comparisons were made of EB1089 vs EB1089 + 9cRA and CB1093 vs CB1093 + 9cRA

vitamin D compounds indicate that the story is more complex and antagonistic as well as synergistic or additive effects can be seen in different systems (discussed in Haussler et al, 1998).

In the present study, the inhibitory effects of ATRA, 9cRA and the vitamin D analogues EB1089 and CB1093 on the three pancreatic adenocarcinoma cell lines AsPc-1, BxPc-3 and T3M-4 were investigated. Our results demonstrate varying degrees of sensitivity to all compounds, as evidenced by inhibition of in vitro growth. However, we also show that the retinoids and the vitamin D analogues employ different inhibitory mechanisms, as 9cRA but neither EB1089 nor CB1093 induced apoptosis and the vitamin D compounds even blocked apoptosis induced by 9cRA in the pancreatic adenocarcinoma cells.

### MATERIALS AND METHODS

### Cell lines

Human pancreatic adenocarcinoma cell lines AsPc-1 (Chen et al, 1982), BxPc-3 (Tan et al, 1986) and T3M-4 (Okabe et al, 1983) were obtained from Prof. N Lemoine (ICRF, London) and the American Tissue Culture Collection. All cells were maintained in RPMI-1640 culture medium with 10% fetal calf serum (FCS), 2 mM L-glutamine and antibiotics at 37°C in a humidified atmosphere containing 5% carbon dioxide. For growth inhibition and apoptosis studies cells were grown in medium containing 2.5% FCS. All cells were routinely tested for mycoplasma contamination.

### Compounds

ATRA and 9cRA were purchased from Sigma (Poole, UK). EB1089 and CB1093 were provided by Leo Pharmaceutical Products (Ballerup, Denmark). The compounds were dissolved in ethanol and stored in the dark at –20°C. Dilutions were made up in complete culture medium and the final ethanol concentration did not exceed 0.1%. The maximal concentrations used were 500 nM for 9cRA and 50 nM for EB 1089 and CB1093.

### Growth inhibition assays

Total cell numbers were assessed using the aminoxanthene dye sulphorhodamine B (SRB). SRB binds to basic amino acid residues in the cells and gives an index of culture cell protein that is linear with cell number (Skehan et al, 1990). Cells were plated in 48-well plates at a density of $2 \times 10^3$ cells per well. After 24–48 h normal culture medium was exchanged for medium containing 2.5% serum and the inhibitory compounds. Control cells received 0.1% ethanol and fresh medium was added every 2–3 days. At the end of each experiment cells were fixed in 10% trichloroacetic acid (TCA) and stained with SRB. Bound SRB was solubilized in unbuffered 10 mM Tris, 50 µl aliquots were transferred to 96-well plates and the optical density was measured at 550 nm.

### Propidium iodide staining and cell cycle analysis

After treatment with 9cRA or EB1089 cells were harvested by trypsinization, washed twice with sample buffer (PBS + 1 g $l^{-1}$ glucose) and fixed in 70% ethanol at a density of $1 \times 10^6$ cells $ml^{-1}$. After > 18 h the cells were washed with sample buffer and resuspended in propidium iodide (PI) staining solution containing 50 µg $ml^{-1}$ PI and 20 µg $ml^{-1}$ RNAase. Fluorescence was measured on a Becton-Dickinson FACScan and DNA histograms were analysed using ModFitLT software.

### Apoptosis assays

The Boehringer-Mannheim Cell Death Detection ELISA kit, which detects the presence of histone-associated DNA fragments in the cell cytosol, was used according to the manual supplied by the manufacturer. Results were confirmed using 7-AAD staining followed by flow cytometry, as previously described (Philpott *et al*, 1996) and PI staining as described above. The three methods were compared and gave very similar results.

### Statistical analysis

All statistical comparisons were made using an unpaired, two-tailed *t*-test; *$P < 0.05$, ** $P < 0.01$, *** $P < 0.005$.

### RESULTS

### Growth inhibition

AsPc-1, BxPc-3 and T3M-4 cells were treated with the test compounds at the indicated concentrations for up to 6 days. Total

© 2000 Cancer Research Campaign

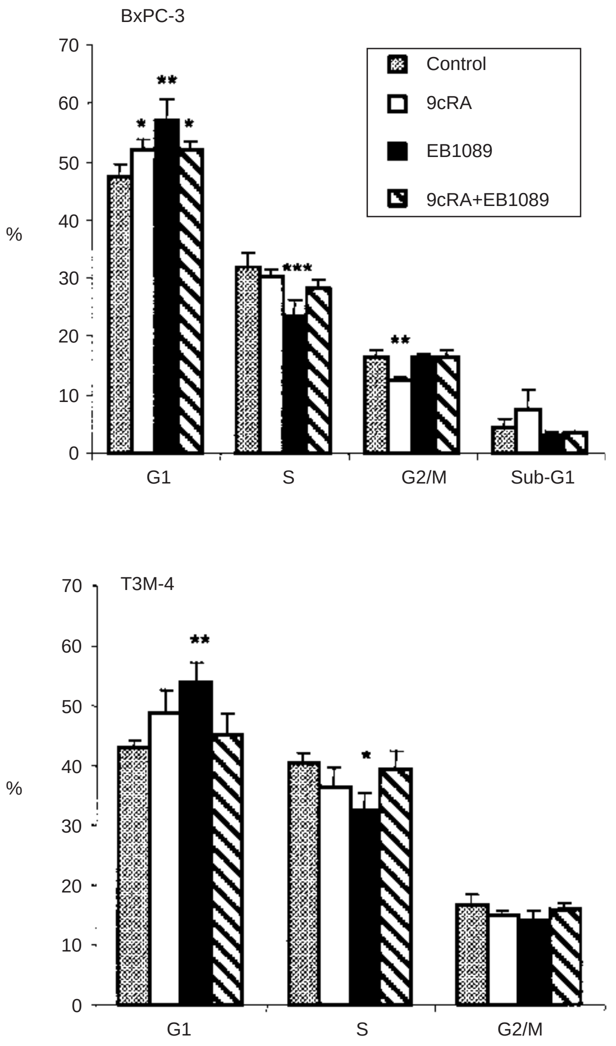

**Figure 3** Growth inhibition was accompanied by an accumulation of cells in the G1 phase of the cell cycle, as determined by propidium iodide staining and flow cytometric analysis. The concentrations used were 500 nM 9cRA and 50 nM EB1089, alone or in combination. Percentages of cell in the different cell cycle phases are shown for BxPc-3 and T3M-4 cells on day 2, and are expressed as means of three determinations cell numbers were assessed using SRB staining. 9cRA, EB1089 as well as CB1093 inhibited growth of the three pancreatic cell lines in vitro, although AsPc-1 cells were less sensitive than BxPc-3 and T3M-4. Reduction in cell numbers as compared to untreated control cells was generally significant from day 4 (not shown). The vitamin D compounds were more potent growth inhibitors than the retinoic acids, with the maximal concentration of 50 nM resulting in stronger inhibition than 50 or 500 nM of 9cRA. They were also significantly more potent on their own than when combined with 9cRA. In two of the cell lines (BxPc-3 and AsPc-1) the combinations were less efficient than any single-agent treatment, whereas in T3M-4 cells the combinations were more efficient than 9cRA on its own but less efficient than EB1089 and CB1093 (Figure 2).

Cell cycle analysis was performed on days 1, 2, 3 and 6 of the treatment period for cells receiving 500 nM 9cRA, 50 nM EB1089 or a combination of the two. An increased proportion of cells in the G0/G1 phase of the cell cycle was observed from day 2, but this increase, together with a decrease in S phase, was statistically significant only in cells receiving EB1089 alone (Figure 3). In cells treated with 9cRA a population of cells containing less DNA than cells in G1 could also be seen, indicating that the cells were undergoing apoptosis (see below). In BxPc-3 cells, a sub-G1 peak could be seen as early as day 2, whereas in AsPc-1 and T3M-4 this change was only detectable on day 6.

Cells treated with the vitamin D analogues displayed characteristic morphological changes. As previously described for butyrate-induced differentiation in pancreatic tumour lines, cells became enlarged and flattened with filamentous protrusions bridging separate cells, indicating that the cells may be undergoing differentiation (E1-Deriny et al, 1987) (Figure 4).

### Induction of apoptosis

Following single-agent treatment with 9cRA, EB1089 or CB1093, or co-treatment with 9cRA + EB1089 or 9cRA + CB1093 for 6 days, apoptosis was assessed as described in Materials and Methods. 9cRA alone was shown to induce apoptosis in all three cell lines in a dose-dependent manner, whereas neither of the two vitamin D analogues had this effect, even at the maximal concentration of 50 nM (Figure 5). When EB1089 or CB1093 were added to T3M-4 cells together with 9cRA a significant decrease in apoptotic cell death could be seen (Figure 5B), indicating that the vitamin D analogues blocked induction of apoptosis by 9cRA. Similar effects were observed in cells co-treated with ATRA + EB1089, such that EB1089 had a clearly negative effect on induction of apoptosis by ATRA (not shown).

T3M-4 cells were subsequently subjected to sequential treatment with 50 nM of EB1089 for three days followed by 500 nM of 9cRA for 6 days. This resulted in complete blockage of apoptosis. In contrast, pretreatment with 9cRA for three days before addition of EB1089 did not make any difference compared to treatment with EB1089 alone (Figure 5C).

Pretreatment with EB1089 for three days also resulted in reduced apoptosis in response to the alkylating agent cisplatin (10 μM for 24 h), showing that the effect was not entirely specific for retinoid-induced apoptosis (Figure 6).

### DISCUSSION

The cell lines AsPc-1, BxPc-3 and T3M-4 are well-established in vitro models of ductal pancreatic adenocarcinoma, representing malignant cells derived from a primary tumour (BxPc-3), a lymph node metastasis (T3M-4) and ascites (AsPc-1). In all three lines, ATRA, 9cRA, EB1089 and CB1093 caused inhibition of cell growth in vitro. Despite this, co-treatment with 9cRA and a vitamin D analogue resulted in inhibition that was significantly weaker than that induced by EB1089 or CB1093 alone. In two of the cell lines the combinations were also less potent than 9cRA on its own. This suggests that the retinoic acids and the vitamin D compounds use different mechanisms to achieve the decrease in cell number seen at the end of the treatment period.

While the retinoic acids were shown to induce apoptotic cell death, EB1089 and CB1093 failed to do so. This is an interesting observation as the cells do express VDR (Kawa et al, 1996 and unpublished data) and are clearly responsive to the analogues, which have been shown to induce apoptosis in various other cell types (James et al, 1995, 1998; Danielsson et al, 1997). Furthermore, EB1089 and CB1093 not only failed to induce apoptosis by themselves, but were also able to completely block

© 2000 Cancer Research Campaign

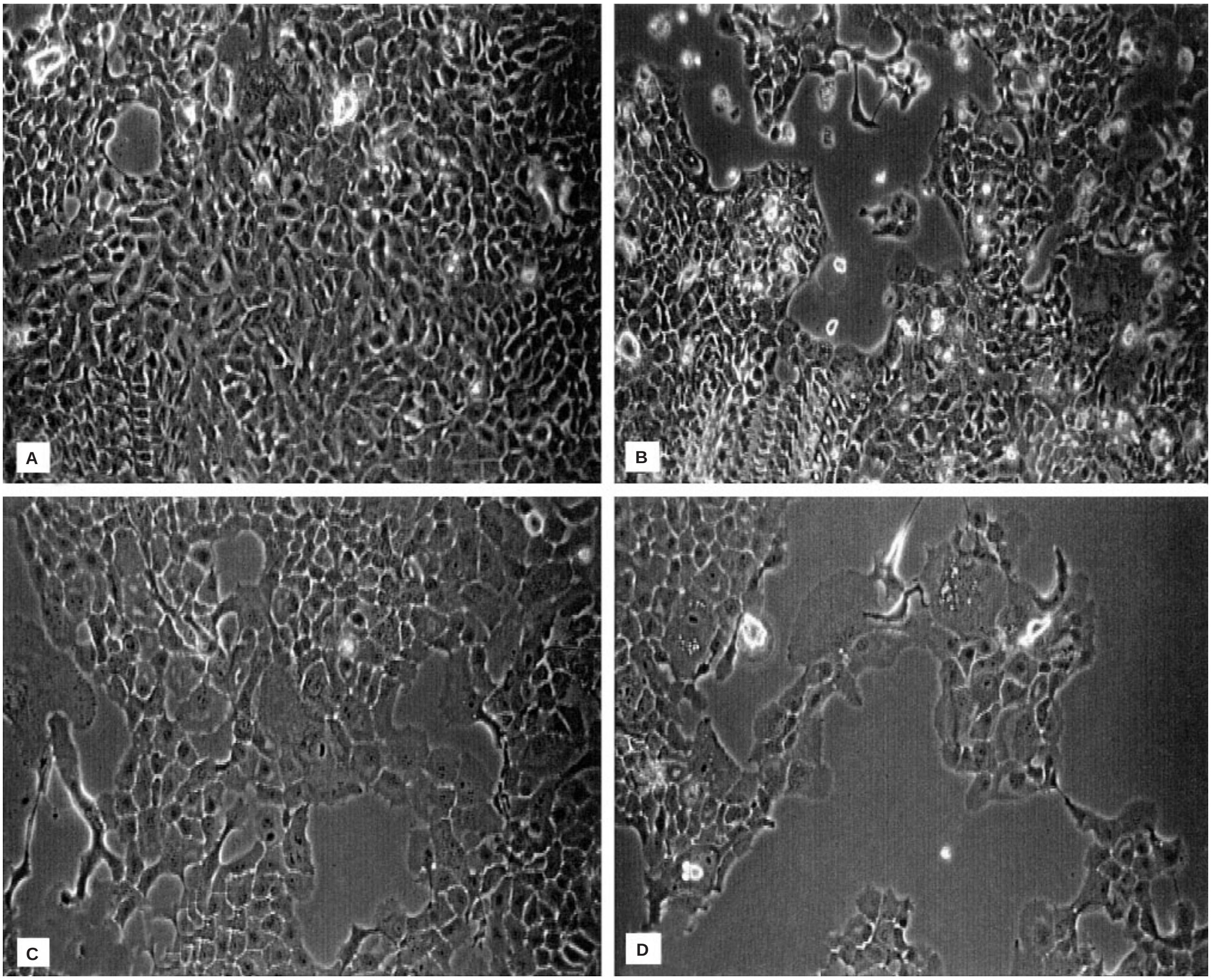

**Figure 4** T3M-4 cells photographed on day 6. Control cells (**A**) have just reached confluency whereas cells treated with 9cRA (**B**) show signs of dying and cells treated with vitamin D analogues (**C, D**) are strongly growth inhibited and display morphological changes induction of apoptosis by 9cRA or ATRA. Again, this suggests that the retinoic acids and the vitamin D compounds activate separate mechanistic pathways and that those pathways are antagonistic and competitive.

The potential mechanisms underlying the antagonism seen between 9cRA and the vitamin D analogues are numerous. Clearly, in the case of co-treatment, direct receptor interactions may play a role. In order to bind efficiently to their specific hormone response elements VDR as well as RARs have to form heterodimers with RXRs. RXRs on the other hand bind to DNA as homodimers. This forms the basis for interactions and competition between pathways activated by retinoids and vitamin D compounds.

Pretreatment with EB1089 for 3 days was shown to be enough to completely block induction of apoptosis by 9cRA. Hence, in this period the vitamin D analogue provoke changes in the cell so that it remains resistant to retinoid-induced apoptosis even after the analogue is removed. Importantly, pretreatment with EB1089 also resulted in reduced apoptosis in response to the non-phase-specific drug cisplatin, which indicates that the phenomenon is not specific for apoptosis induced by retinoids, and accordingly effects on RAR/RXR expression or receptor interactions cannot be solely responsible. In agreement with this, assessment of receptor expression levels during different treatments showed no evidence of any regulation that could be responsible for the observed antagonism (not shown). It is therefore likely that regulation by vitamin D analogues of genes whose products are involved in cell differentiation, proliferation and death play the most important role in this resistance. A number of genes are regulated by $1,25D_3$ and its analogues, including the cyclin-dependent kinase inhibitors (CDKI) $p21^{WAF-1}$ and $p27^{Kip1}$ (Liu et al, 1996; Wang et al, 1996; Matsumoto et al, 1998). Both these proteins have been shown to be up-regulated in the pancreatic cancer cell line BxPc-3 after treatment with $1,25D_3$ as well as the synthetic analogue 22-oxa-1,25-dihydroxyvitamin $D_3$ (OCT) (Kawa et al, 1997), implying that this is the mechanism whereby G0/G1 arrest is achieved. $p21^{WAF-1}$ can also protect cells from apoptosis in some systems (Gartel and Tyner, 1999).

It has already been established that different cell types, and even different cell lines of the same type, respond in different ways to retinoids, vitamin D compounds and combinations of the two. Additive and synergistic effects of the two groups of compounds

 *© 2000 Cancer Research Campaign*

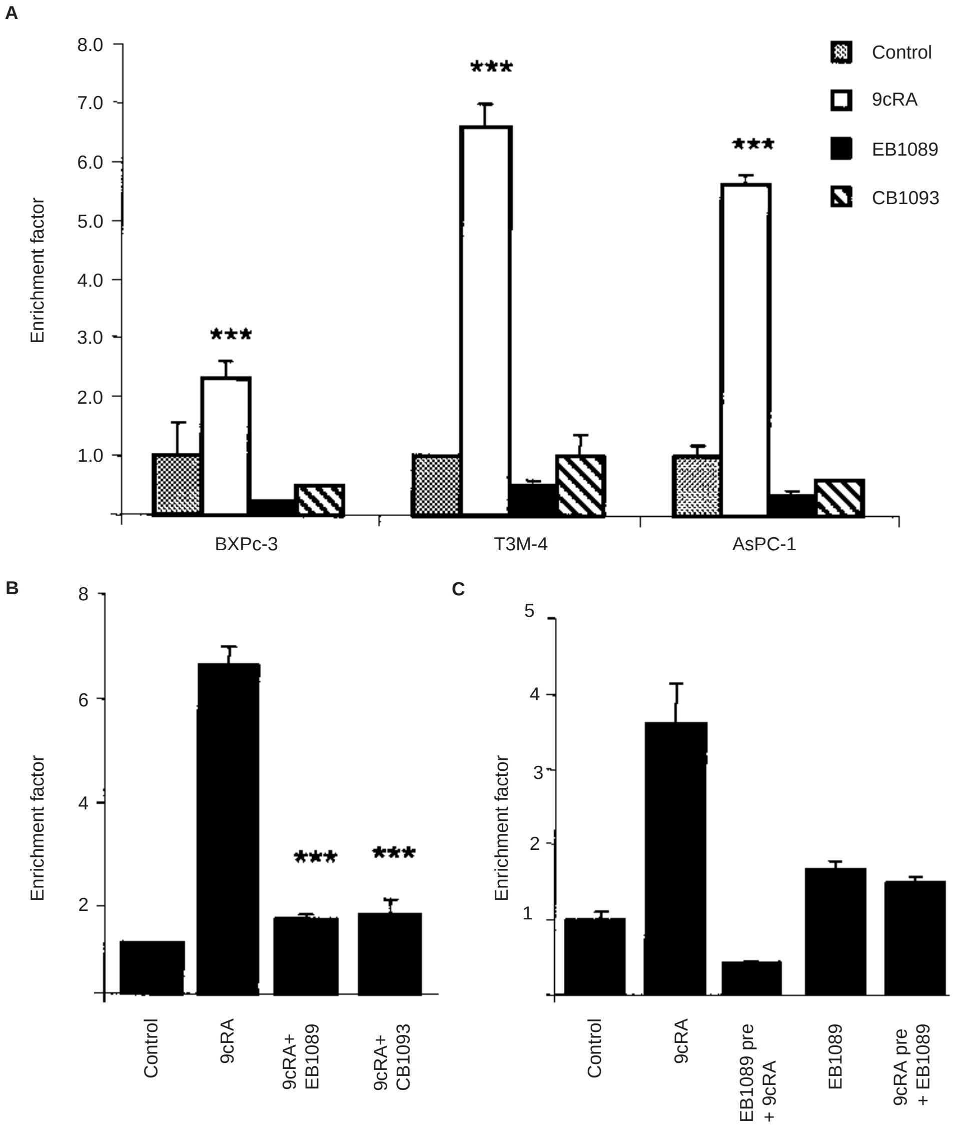

**Figure 5** Assessment of apoptosis by Cell Death Detection ELISA, detecting the presence of histone-associated DNA fragments in the cell cytosol. The enrichment factor is calculated as $OD_{(treated\ cells)}/OD_{(untreated\ cells)}$. (**A**) 9cRA, but neither EB1089 nor CB1093 induced apoptosis in the three cell lines. (**B**) Combinations of 9cRA with EB1089 or CB1089 resulted in very weak induction of apoptosis in T3M-4 cells compared to treatment with 9cRA alone, and (**C**) preincubation with EB1089 for 3 days completely blocked induction of apoptosis by 9cRA. The concentrations used were 500 nM of 9cRA and 50 nM of the vitamin D analogues. Statistical comparisons were made of control vs treated cells (**A**) and 9cRA vs 9cRA + EB1089 or CB1093 (**B**)

are commonly seen, e.g. in breast and prostate cancer cells where both groups induce growth inhibition, G0/G1 arrest and apoptosis (James et al, 1995; Elstner et al, 1999; Koshizuka et al, 1999). Leukaemia cells on the other hand, show a different response pattern. 9cRA as well as well as vitamin D induce differentiation, and do so in a cooperative manner. Furthermore, it has been observed in HL-60 cells that vitamin D derivatives, although unable to induce apoptosis on their own, can potentiate induction of apoptosis by 9cRA (James et al, 1999). Differential effects have also been shown in melanoma and colon cancer cells (Kane et al, 1996; Danielsson et al, 1999).

In pancreatic cancer cells, responsiveness to both retinoids and vitamin D analogues have been reported previously (Rosewicz et al, 1995; Bold et al, 1996; Kawa et al, 1996; Louvet et al, 1996; Zugmaier et al, 1996; Colston et al, 1997). However, those studies have mainly assessed inhibition of cell growth and apoptosis has not been examined. In only one study were the combined effects of retinoic acid and a vitamin D derivative studied (Zugmaier et al, 1996), and in contrast to our results, EB1089 potentiated the growth inhibitory effects of both ATRA and 9cRA. The reason for this divergent effect, is most likely that the cell lines studied (Capan-1 and -2) are at a different stage of differentiation compared to our cell lines and distinct expression patterns of RARs, RXRs and VDR may account for contrasting responses. Capan-1 and -2 express lower levels of VDR than *e.g.* BxPc-3 and are significantly less sensitive to $1,25D_3$ than this cell line (Kawa et al, 1996). RAR-α and γ are expressed in our cell lines as well as Capan-1 and -2, but no or low levels of RAR-β are generally detected (Rosewicz et al, 1995; Kaiser et al, 1998 and our observations).

*© 2000 Cancer Research Campaign* 

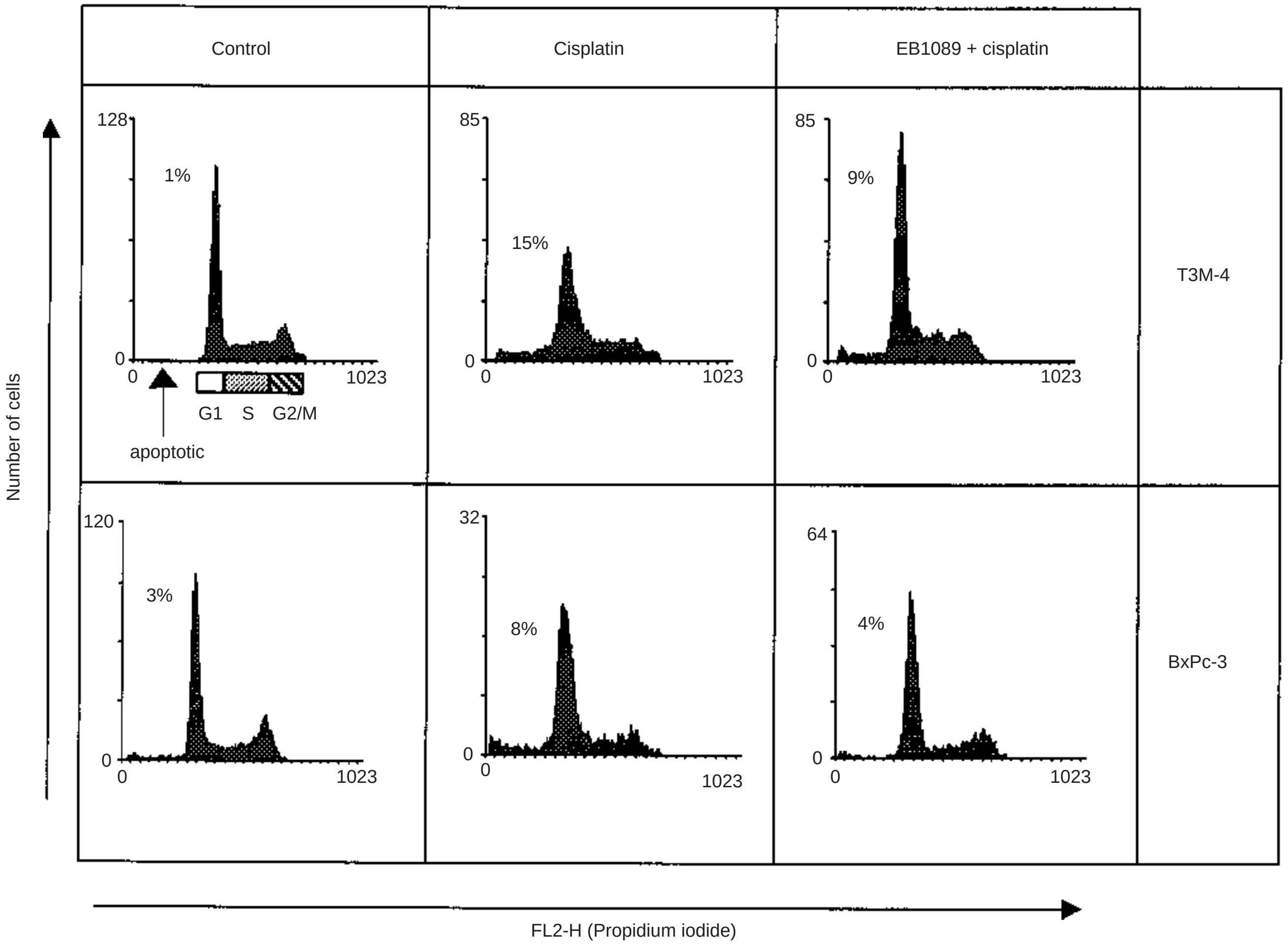

**Figure 6** Propidium iodide staining and flow cytometric analysis of T3M-4 and BxPc-3 cells showed that pretreatment with EB1089 for 3 days before addition of cisplatin (10 μM) for 24 h reduced the percentage of cells with sub-G1 DNA content. Percentages shown are means of two determinations. The total number of cells being analysed varies between the samples, as cell fragments and aggregates of two or more cells had to be excluded

The Bcl-2 family of proteins regulates responses to a variety of apoptotic stimuli, and includes anti-apoptotic (Bcl-2, Bcl-$X_L$, Mcl-1 and A1) as well as pro-apoptotic members (Bax, Bcl-$X_s$ etc.) (Korsmeyer, 1999). For example, in breast cancer cells which undergo apoptosis in response to vitamin D compounds, down-regulation of the anti-apoptotic protein Bcl-2 and an increased Bax/Bcl-2 ratio can be observed (James *et al*, 1998). This is also observed in HL-60 leukaemia cells, and is thought to be a mechanism whereby vitamin D derivatives potentiate induction of apoptosis by 9cRA (James *et al*, 1999). However, 1,25$D_3$ has also been reported to have an anti-apoptotic effect in HL-60 cells, and this was associated with induction of differentiation and up-regulation of Mcl-1 (Wang and Studzinski, 1997). We are currently investigating the effects of EB1089, CB1093 and 9cRA on expression of these pro- and anti-apoptotic proteins in pancreatic cancer cells, to assess their role in the differential effects observed.

The conclusions that can be drawn from this study are that both retinoids and vitamin D analogues may have a role to play in management of pancreatic cancer, but the outcome of the two treatments will be different. Vitamin D analogues can slow down cell growth but do not kill the cells, whereas retinoids do both. It also points out that the two groups of compounds should probably not be used together as they counteract each other in vitro.

### ACKNOWLEDGEMENTS

This work was supported by The Ralph Bates Pancreatic Research Fund.

### REFERENCES

Bold RJ, Ishizuka J, Townsend CMJ and Thompson JC (1996) All-trans-retinoic acid inhibits growth of human pancreatic cancer cell lines. *Pancreas* **12**: 189–195

Bollag W, Majewski S and Jablonska S (1994) Cancer combination chemotherapy with retinoids: experimental rationale. *Leukemia* **8**: 1453–1457

Chen WH, Horoszewicz JS, Leong SS, Shimano T, Penetrante T, Sanders WH, Berjian R, Douglass HO, Martin EW and Ming Chu T (1982) Human pancreatic adenocarcinoma: in vitro and in vivo morphology of a new tumor line established from ascites. *In vitro* **18**: 24–34

Colston KW (1997) Vitamin D and breast cancer: therapeutic potential of new vitamin D analogues. In: *Vitamin D*, Feldman D (ed), pp. 1107–1123. Academic Press, San Diego

Colston KW, James SJ, Ofori-Kuragu EA, Binderup L and Grant AG (1997) Vitamin D receptors and anti-proliferative effects of vitamin D derivatives in human pancreatic carcinoma cells in vivo and in vitro. *Br J Cancer* **76**: 1017

Danielsson C, Mathiasen IS, James SJ, Nayeri S, Bretting C, Hansen CM, Colston KW and Carlberg C (1997) Sensitive induction of apoptosis in breast cancer cells by a novel 1,25-dihydroxyvitamin D3 analogue shows relation to promoter selectivity. *J Cell Biochem* **66**: 552–562

 © 2000 Cancer Research Campaign

Danielsson C, Torma H, Vahlquist A and Carlberg C (1999) Positive and negative interaction of 1,25-dihydroxyvitamin D3 and the retinoid CD437 in the induction of human melanoma cell apoptosis. *Int J Cancer* **81**: 467–470

Dippold W, Bernhard H, Meyer zum B and schenfelde KH (1997) Chemotherapy in advanced pancreatic cancer. *Int J Pancreatol* **21**: 39–41

El-Deriny S, O'Brien MJ, Christensen TG, and Kupchik HZ (1987) Ultrastructural differentiation and CEA expression of butyrate-treated human pancreatic carcinoma cells. *Pancreas* **2**: 25–33

Elstner E, Campbell MJ, Munker R, Shintaku P, Binderup L, Heber D, Said J and Koeffler HP (1999) Novel 20-epi-vitamin D analog combined with 9-*cis*-retinoic acid markedly inhibits colony growth of prostate cancer cells. *Prostate* **40**: 141–149

Evans RM (1998) The steroid and thyroid hormone receptor superfamily. *Science* **240**: 889–895

Evans TRJ, Lofts FJ, Mansi JL, Glees JP, Dalgleish AG and Knight MJ (1996) A phase II study of continuous-infusion 5-fluorouracil with cisplatin and epirubicin in inoperable pancreatic cancer. *Br J Cancer* **73**: 1260–1264

Gansauge S, Gansauge F and Beger HG (1996) Molecular oncology in pancreatic cancer. *J Mol Med* **74**: 313–320

Gartel AL and Tyner AL (1999) Transcriptional regulation of the p21$^{(WAF1/CIP1)}$ gene. *Exp Cell Res* **246**: 280–289

Giguere V, Ong ES, Segui P and Evans RM (1987) Identification of a receptor for the morphogen retinoic acid. *Nature* **330**: 624–629

Haussler MR, Jurutka PW, Hsieh J-C, Thompson PD, Selznick SH, Haussler CA and Whitfield GK (1995) New understanding of the molecular mechanisms of receptor-mediated genomic actions of the vitamin D hormone. *Bone* **17**: 33S–38S

Haussler MR, Whitfield GK, Haussler CA, Hsieh JC, Thompson PD, Selznick SH, Dominguez CE and Jurutka PW (1998) The nuclear vitamin D receptor: Biological and molecular regulatory properties revealed. *J Bone Min Res* **13**: 325–342

Heyman RA, Mangelsdorf DJ, Dyck JA, Stein RB, Eichele G, Evans RM and Thaller C (1992) 9-cis retinoic acid is a high affinity ligand for the retinoid X receptor. *Cell* **68**: 397–406

James SY, Mackay AG and Colston KW (1995) Vitamin D derivatives in combination with 9-cis-retinoic acid promote active cell death in breast cancer cells. *J Mol Endocrinol* **14**: 391–394

James SY, Mercer E, Brady M Binderup L and Colston KW (1998) EB1089, a synthetic analogue of vitamin D, induces apoptosis in breast cancer cells in vivo and in vitro. *Br J Pharmacol* **125**: 953–962

James SY, Williams MA, Newland AC and Colston KW (1999) Leukemia cell differentiation: cellular and molecular interactions of retinoids and vitamin D. *Gen Pharmacol* **32**: 143–154

Kaiser A, Wolf-Breitinger M, Albers A, Dorbic T, Wittig B, Riecken EO and Rosewicz S (1998) Retinoic acid receptor gamma-1 expression determines retinoid sensitivity in pancreatic carcinoma cells. *Gastroenterology* **115**: 967–977

Kane KF, Langman MJS and Williams GR (1996) Antiproliferative responses of two human colon cancer cell lines to vitamin D3 are differentially modified by 9-cis-retinoic acid. *Cancer Res* **56**: 623–632

Kawa S, Yoshizawa K, Tokoo M, Imai H, Oguchi H, Kiyosawa K, Homma T, Nikaido T and Furihata K (1996) Inhibitory effect of 22-oxa-1,25-dihydroxyvitamin D3 on the proliferation of pancreatic cancer cell lines. *Gastroenterology* **110**: 1605–1613

Kawa S, Nikaido T, Zhai Y, Kumagai T, Furihata K, Fujii S and Kiyosawa K (1997) Vitamin D analogues up-regulate p21 and p27 during growth inhibition of pancreatic cancer cell lines. *Br J Cancer* **76**: 884–889

Kliewer SA, Umesono K, Mangelsdorf DJ and Evans RM (1992) Retinoid X receptor interacts with nuclear receptors in retinoic acid, thyroid hormone and vitamin D3 signalling. *Nature* **355**: 446–449

Korsmeyer SJ (1999) BCL-2 gene family and the regulation of programmed cell death. *Cancer Res* **59**: 1693s–1700s

Koshizuka K, Kubota T, Said J, Koike M, Binderup L, Uskokovic M and Koeffler HP (1999) Combination therapy of a vitamin D analog and all-trans-retinoic acid: effect on human breast cancer in nude mice. *Anticancer Res* **19**: 519–524

Levin AA, Sturzenbecker LJ, Kazmer S, Bosakowski T, Huselton C, Allenby G, Speck G, Kratzeisen C, Rosenberger M, Lovey and A Grippo JF (1992) 9-cis retinoic acid stereoisomer binds and activates the nuclear receptor RXRα. *Nature* **355**: 359–361

Liu M, Lee M-H, Cohen M, Bommakanti M and Freedman LP (1996) Transcriptional activation of the Cdk inhibitor p21 by vitamin D leads to the induced differentiation of the myelomonocytic cell line U937. *Genes Dev* **10**: 142–153

Lotan R (1996) Retinoids in cancer chemoprevention. *Faseb J* **10**: 1031–1039

Louvet C, Djelloul S, Forgue-Lafitte ME, Mester J, Zimber A and Gespach C (1996) Antiproliferative effects of the arotinoid Ro 40-8757 in human gastrointestinal and pancreatic cancer cell lines: combinations with 5-fluorouracil and interferon-alpha. *Br J Cancer* **74**: 394–399

Mathiasen IS, Colston KW and Binderup L (1993) EB1089, a novel vitamin D analogue, has strong antiproliferative and differentiation inducing effects on cancer cells. *J Steroid Biochem Mol Biol* **46**: 365–371

Matsumoto T, Sowa Y, Ohtani-Fujita N, Tamaki T, Takenaka T, Kuribayashi K and Sakai T (1998) p53-independent induction of WAF1/Cip1 is correlated with osteoblastic differentiation by vitamin D3. *Cancer Lett* **129**: 61–68

Nagy L, Thomazy VA, Heyman RA and Davies PJ (1998) Retinoid-induced apoptosis in normal and neoplastic tissues. *Cell Death Differ* **5**: 11–19

Okabe T, Yamaguchi N and Oshawa N (1983) Establishment and characterization of a carcinoembryonic antigen (CEA)-producing cell line from a human carcinoma of the exocrine pancreas. *Cancer* **51**: 662–668

Petkovich M, Brand NJ, Krust A and Chambon P (1987) A human retinoic acid receptor which belongs to the family of nuclear receptors. *Nature* **330**: 444–450

Philpott NJ, Turner AJ, Scopes J, Westby M, Marsh JC, Gordon-Smith EC, Dalgleish AG and Gibson AG (1996) The use of 7-amino actinomycin D in identifying apoptosis: simplicity of use and broad spectrum of application compared with other techniques. *Blood* **87**: 2244–2251

Rosewicz S and Wiedenmann B (1997) Pancreatic carcinoma. *Lancet* **349**: 485–489

Rosewicz S, Stier U, Brembeck F, Wiedenmann B and Riecken EO (1995) Retinoids: effect on growth, differentiation and nuclear receptor expression in human pancreatic carcinoma cell lines. *Gastroenterology* **109**: 1646–1660

Rothenberg ML, Moore MJ, Cripps MC, Andersen, Portenoy RK, Burris HA, Green MR, Tarassoff PG, Brown TD, Casper ES, Stornioli AM and Von Hoff DD (1996) A phase II trial of gemcitabine in patients with 5-FU-refractory pancreas cancer. *Ann Oncol* **7**: 247–253

Skehan P, Storeng R, Scudiero D, Monks A, McMahon J, Vistica D, Warren JT, Bokesh H, Kenney S and Boyd MR (1990) New colorimetric cytotoxicity assay for anticancer-drug screening. *J Natl Cancer Inst* **82**: 1107–1112

Tan MH, Nowak NJ, Loor R, Ochi H, Sandberg AA, Lopez C, Pickren JW, Berjian, R, Douglass HD and Chu TM (1986) Characterization of a new primary human pancreatic tumour line. *Cancer Invest* **4**: 15–23

Wang QM, Jones JB and Studzinski GP (1996) Cyclin-dependent kinase inhibitor p27 as a mediator of the G1-S phase block induced by 1,25-dihydroxyvitamin D3 in HL60 cells. *Cancer Res* **56**: 264–267

Wang X and Studzinski GP (1997) Antiapoptotic action of 1,25-dihydroxyvitamin D3 is associated with increased mitochondrial MCL-1 and RAF-1 proteins and reduced release of cytochrome c. *Exp Cell Res* **235**: 210–217

Zugmaier G, Jager R, Grage B, Gottardis MM, Havemann K and Knabbe C (1996) Growth-inhibitory effects of vitamin D analogues and retinoids on human pancreatic cancer cells. *Br J Cancer* **73**: 1341–1346

*© 2000 Cancer Research Campaign*

## References

[PDF_00482] Bold R. J., Ishizuka J., Townsend C. M., Thompson J. C. (1996). All-trans-retinoic acid inhibits growth of human pancreatic cancer cell lines.. Pancreas.

[PDF_00485] Bollag W., Majewski S., Jablonska S. (1994). Cancer combination chemotherapy with retinoids: experimental rationale.. Leukemia.

[PDF_00487] Chen W. H., Horoszewicz J. S., Leong S. S., Shimano T., Penetrante R., Sanders W. H., Berjian R., Douglass H. O., Martin E. W., Chu T. M. (1982). Human pancreatic adenocarcinoma: in vitro and in vivo morphology of a new tumor line established from ascites.. In Vitro.

[PDF_00494] Colston K. W., James S. Y., Ofori-Kuragu E. A., Binderup L., Grant A. G. (1997). Vitamin D receptors and anti-proliferative effects of vitamin D derivatives in human pancreatic carcinoma cells in vivo and in vitro.. Br J Cancer.

[PDF_00497] Danielsson C., Mathiasen I. S., James S. Y., Nayeri S., Bretting C., Hansen C. M., Colston K. W., Carlberg C. (1997). Sensitive induction of apoptosis in breast cancer cells by a novel 1,25-dihydroxyvitamin D3 analogue shows relation to promoter selectivity.. J Cell Biochem.

[PDF_00533] Danielsson C., Törmä H., Vahlquist A., Carlberg C. (1999). Positive and negative interaction of 1,25-dihydroxyvitamin D3 and the retinoid CD437 in the induction of human melanoma cell apoptosis.. Int J Cancer.

[PDF_00536] Dippold W., Bernhard H., Meyer zum Büschenfelde K. H. (1997). Chemotherapy in advanced pancreatic cancer.. Int J Pancreatol.

[PDF_00541] Elstner E., Campbell M. J., Munker R., Shintaku P., Binderup L., Heber D., Said J., Koeffler H. P. (1999). Novel 20-epi-vitamin D3 analog combined with 9-cis-retinoic acid markedly inhibits colony growth of prostate cancer cells.. Prostate.

[PDF_00545] Evans R. M. (1988). The steroid and thyroid hormone receptor superfamily.. Science.

[PDF_00547] Evans T. R., Lofts F. J., Mansi J. L., Glees J. P., Dalgleish A. G., Knight M. J. (1996). A phase II study of continuous-infusion 5-fluorouracil with cisplatin and epirubicin in inoperable pancreatic cancer.. Br J Cancer.

[PDF_00550] Gansauge S., Gansauge F., Beger H. G. (1996). Molecular oncology in pancreatic cancer.. J Mol Med (Berl).

[PDF_00552] Gartel A. L., Tyner A. L. (1999). Transcriptional regulation of the p21((WAF1/CIP1)) gene.. Exp Cell Res.

[PDF_00555] Giguere V., Ong E. S., Segui P., Evans R. M. (1987). Identification of a receptor for the morphogen retinoic acid.. Nature.

[PDF_00557] Haussler M. R., Jurutka P. W., Hsieh J. C., Thompson P. D., Selznick S. H., Haussler C. A., Whitfield G. K. (1995). New understanding of the molecular mechanism of receptor-mediated genomic actions of the vitamin D hormone.. Bone.

[PDF_00561] Haussler M. R., Whitfield G. K., Haussler C. A., Hsieh J. C., Thompson P. D., Selznick S. H., Dominguez C. E., Jurutka P. W. (1998). The nuclear vitamin D receptor: biological and molecular regulatory properties revealed.. J Bone Miner Res.

[PDF_00565] Heyman R. A., Mangelsdorf D. J., Dyck J. A., Stein R. B., Eichele G., Evans R. M., Thaller C. (1992). 9-cis retinoic acid is a high affinity ligand for the retinoid X receptor.. Cell.

[PDF_00568] James S. Y., Mackay A. G., Colston K. W. (1995). Vitamin D derivatives in combination with 9-cis retinoic acid promote active cell death in breast cancer cells.. J Mol Endocrinol.

[PDF_00571] James S. Y., Mercer E., Brady M., Binderup L., Colston K. W. (1998). EB1089, a synthetic analogue of vitamin D, induces apoptosis in breast cancer cells in vivo and in vitro.. Br J Pharmacol.

[PDF_00574] James S. Y., Williams M. A., Newland A. C., Colston K. W. (1999). Leukemia cell differentiation: cellular and molecular interactions of retinoids and vitamin D.. Gen Pharmacol.

[PDF_00577] Kaiser A., Wolf-Breitinger M., Albers A., Dorbic T., Wittig B., Riecken E. O., Rosewicz S. (1998). Retinoic acid receptor gamma1 expression determines retinoid sensitivity in pancreatic carcinoma cells.. Gastroenterology.

[PDF_00581] Kane K. F., Langman M. J., Williams G. R. (1996). Antiproliferative responses to two human colon cancer cell lines to vitamin D3 are differently modified by 9-cis-retinoic acid.. Cancer Res.

[PDF_00588] Kawa S., Nikaido T., Aoki Y., Zhai Y., Kumagai T., Furihata K., Fujii S., Kiyosawa K. (1997). Vitamin D analogues up-regulate p21 and p27 during growth inhibition of pancreatic cancer cell lines.. Br J Cancer.

[PDF_00584] Kawa S., Yoshizawa K., Tokoo M., Imai H., Oguchi H., Kiyosawa K., Homma T., Nikaido T., Furihata K. (1996). Inhibitory effect of 220-oxa-1,25-dihydroxyvitamin D3 on the proliferation of pancreatic cancer cell lines.. Gastroenterology.

[PDF_00591] Kliewer S. A., Umesono K., Mangelsdorf D. J., Evans R. M. (1992). Retinoid X receptor interacts with nuclear receptors in retinoic acid, thyroid hormone and vitamin D3 signalling.. Nature.

[PDF_00594] Korsmeyer S. J. (1999). BCL-2 gene family and the regulation of programmed cell death.. Cancer Res.

[PDF_00596] Koshizuka K., Kubota T., Said J., Koike M., Binderup L., Uskokovic M., Koeffler H. P. (1999). Combination therapy of a vitamin D3 analog and all-trans-retinoic acid: effect on human breast cancer in nude mice.. Anticancer Res.

[PDF_00599] Levin A. A., Sturzenbecker L. J., Kazmer S., Bosakowski T., Huselton C., Allenby G., Speck J., Kratzeisen C., Rosenberger M., Lovey A. (1992). 9-cis retinoic acid stereoisomer binds and activates the nuclear receptor RXR alpha.. Nature.

[PDF_00603] Liu M., Lee M. H., Cohen M., Bommakanti M., Freedman L. P. (1996). Transcriptional activation of the Cdk inhibitor p21 by vitamin D3 leads to the induced differentiation of the myelomonocytic cell line U937.. Genes Dev.

[PDF_00607] Lotan R. (1996). Retinoids in cancer chemoprevention.. FASEB J.

[PDF_00608] Louvet C., Djelloul S., Forgue-Lafitte M. E., Mester J., Zimber A., Gespach C. (1996). Antiproliferative effects of the arotinoid Ro 40-8757 in human gastrointestinal and pancreatic cancer cell lines: combinations with 5-fluorouracil and interferon-alpha.. Br J Cancer.

[PDF_00612] Mathiasen I. S., Colston K. W., Binderup L. (1993). EB 1089, a novel vitamin D analogue, has strong antiproliferative and differentiation inducing effects on cancer cells.. J Steroid Biochem Mol Biol.

[PDF_00615] Matsumoto T., Sowa Y., Ohtani-Fujita N., Tamaki T., Takenaka T., Kuribayashi K., Sakai T. (1998). p53-independent induction of WAF1/Cip1 is correlated with osteoblastic differentiation by vitamin D3.. Cancer Lett.

[PDF_00618] Nagy L., Thomazy V. A., Heyman R. A., Davies P. J. (1998). Retinoid-induced apoptosis in normal and neoplastic tissues.. Cell Death Differ.

[PDF_00620] Okabe T., Yamaguchi N., Ohsawa N. (1983). Establishment and characterization of a carcinoembryonic antigen (CEA)-producing cell line from a human carcinoma of the exocrine pancreas.. Cancer.

[PDF_00623] Petkovich M., Brand N. J., Krust A., Chambon P. (1987). A human retinoic acid receptor which belongs to the family of nuclear receptors.. Nature.

[PDF_00626] Philpott N. J., Turner A. J., Scopes J., Westby M., Marsh J. C., Gordon-Smith E. C., Dalgleish A. G., Gibson F. M. (1996). The use of 7-amino actinomycin D in identifying apoptosis: simplicity of use and broad spectrum of application compared with other techniques.. Blood.

[PDF_00632] Rosewicz S., Stier U., Brembeck F., Kaiser A., Papadimitriou C. A., Berdel W. E., Wiedenmann B., Riecken E. O. (1995). Retinoids: effects on growth, differentiation, and nuclear receptor expression in human pancreatic carcinoma cell lines.. Gastroenterology.

[PDF_00630] Rosewicz S., Wiedenmann B. (1997). Pancreatic carcinoma.. Lancet.

[PDF_00639] Skehan P., Storeng R., Scudiero D., Monks A., McMahon J., Vistica D., Warren J. T., Bokesch H., Kenney S., Boyd M. R. (1990). New colorimetric cytotoxicity assay for anticancer-drug screening.. J Natl Cancer Inst.

[PDF_00642] Tan M. H., Nowak N. J., Loor R., Ochi H., Sandberg A. A., Lopez C., Pickren J. W., Berjian R., Douglass H. O., Chu T. M. (1986). Characterization of a new primary human pancreatic tumor line.. Cancer Invest.

[PDF_00645] Wang Q. M., Jones J. B., Studzinski G. P. (1996). Cyclin-dependent kinase inhibitor p27 as a mediator of the G1-S phase block induced by 1,25-dihydroxyvitamin D3 in HL60 cells.. Cancer Res.

[PDF_00648] Wang X., Studzinski G. P. (1997). Antiapoptotic action of 1,25-dihydroxyvitamin D3 is associated with increased mitochondrial MCL-1 and RAF-1 proteins and reduced release of cytochrome c.. Exp Cell Res.

[PDF_00651] Zugmaier G., Jäger R., Grage B., Gottardis M. M., Havemann K., Knabbe C. (1996). Growth-inhibitory effects of vitamin D analogues and retinoids on human pancreatic cancer cells.. Br J Cancer.

[PDF_00538] el-Deriny S. E., O'Brien M. J., Christensen T. G., Kupchik H. Z. (1987). Ultrastructural differentiation and CEA expression of butyrate-treated human pancreatic carcinoma cells.. Pancreas.

